# Supplementary material for: Foraging Under Uncertainty Follows the Marginal Value Theorem with Bayesian Updating of Environment Representations
Source: bioRxiv. 2024 Mar 31:2024.03.30.587253. Preprint. [Version 1] doi: 10.1101/2024.03.30.587253 (PMC10996644; doi:10.1101/2024.03.30.587253)
Supplement: Supplement 1 [file NIHPP2024.03.30.587253v1-supplement-1.pdf]

bioRxiv preprint doi: https://doi.org/10.1101/2024.03.30.587253; this version posted March 31, 2024. The copyright holder for this preprint (which was not certified by peer review) is the author/funder, who has granted bioRxiv a license to display the preprint in perpetuity. It is made available under aCC-BY 4.0 International license.

# 8 Supplementary Materials

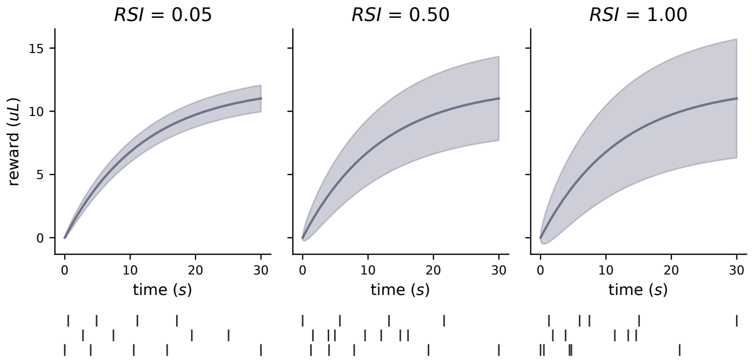


Figure S1: (*top*) Cumulative reward functions for environments with $\tau = 12.0$ and a low (left), moderate (center), or high (right) level of stochasticity in the reward dynamics, shown as mean ($V_0\Lambda(t)$, solid curve) $\pm$ standard deviation ($V_0^2\Lambda(t)$, shaded area). (*bottom*) Three example reward sequences for each level of stochasticity.

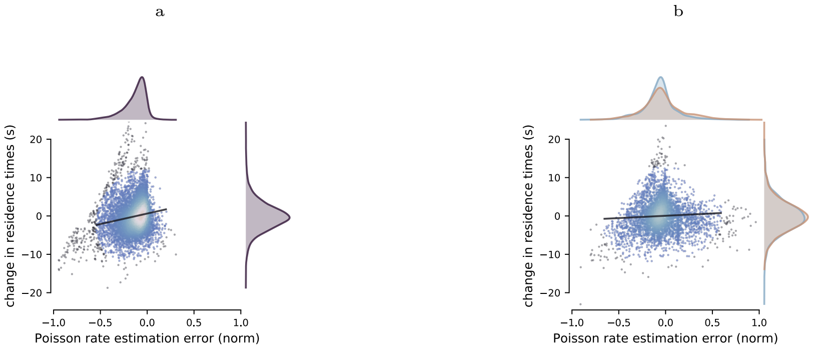


Figure S2: Correlations in local information were not present in shuffled data. Within sessions, residence times were shuffled across patches prior to calculating to rate estimation error at patch-leaving and the change in residence time relative to the session average. The scatter plot, regression line, and marginal distributions were then calculated from the shuffled data as in Figure 4B-C for environments with (**A**) low ($RSI = 0.05$) and (**B**) moderate to high ($RSI \in [0.5, 1.0]$) stochasticity.

bioRxiv preprint doi: https://doi.org/10.1101/2024.03.30.587253; this version posted March 31, 2024. The copyright holder for this preprint (which was not certified by peer review) is the author/funder, who has granted bioRxiv a license to display the preprint in perpetuity. It is made available under aCC-BY 4.0 International license.

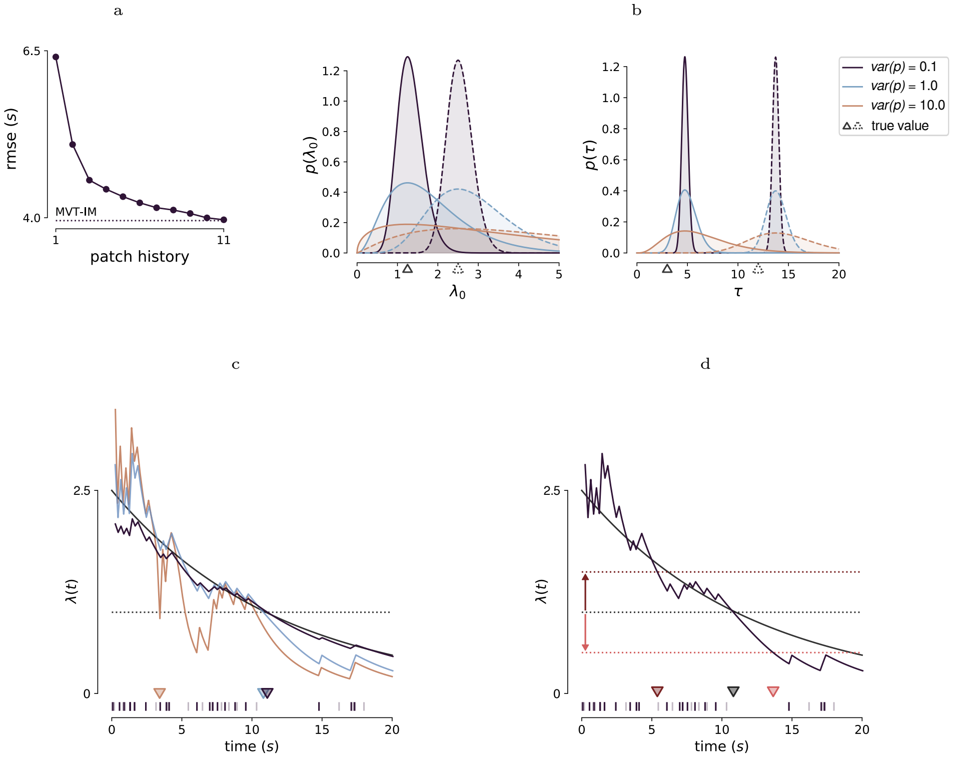


Figure S3: Aspects of the Bayesian behavioral models. (**A**) The root-mean-square prediction errors (RMSE) of various MLE-x models (solid line) were calculated for all patches in environments with moderate-to-high stochasticity ($RSI \in [0.5, 1.0]$). In order to estimate the Poisson rate, models utilized the observations from the current patch plus zero (MLE-1) up to ten (MLE-11) of the previous patch encounters. The prediction error asymptotically approached that of the MVT-IM model (dotted line). (**B**) Two example prior distributions (solid or dotted curve) for the initial Poisson rate ($lambda_0$; left) and decay rate ($\tau$; right) are shown for three different levels of variance ($var(p)$). Prior distributions were generated from a gamma distribution such that the mode was equal to either the experimental ($\lambda_0$) or internally-modeled ($\tau$) value for the environment (solid or dotted triangle). (**C**) The Poisson rate estimates of the MAP-IM-L model are shown for an example reward sequence in a patch (raster at bottom), which consists of unobserved (light purple) and observed (dark purple) events. The rate estimates utilized observations from the example patch and reflect prior distributions with high (orange), moderate (light blue), and low (dark purple) levels of uncertainty, as shown in **B**. The predicted leaving times for the models (colored triangles) occur when the estimated rates fall below a given threshold for the environment (dotted black line) that is derived from the MVT-IM model. (**D**) Given the same example reward sequence as in **C**, the MAP-IM-GL model estimates the Poisson rate (purple solid line) from observations (raster at bottom) and additionally modulates the rate threshold for patch-leaving (black dotted line). Higher (dark red) or lower (light red) thresholds lead to earlier or later leaving times, respectively. The true Poisson rate in **C** and **D** is shown by the black curve.

bioRxiv preprint doi: https://doi.org/10.1101/2024.03.30.587253; this version posted March 31, 2024. The copyright holder for this preprint (which was not certified by peer review) is the author/funder, who has granted bioRxiv a license to display the preprint in perpetuity. It is made available under aCC-BY 4.0 International license.

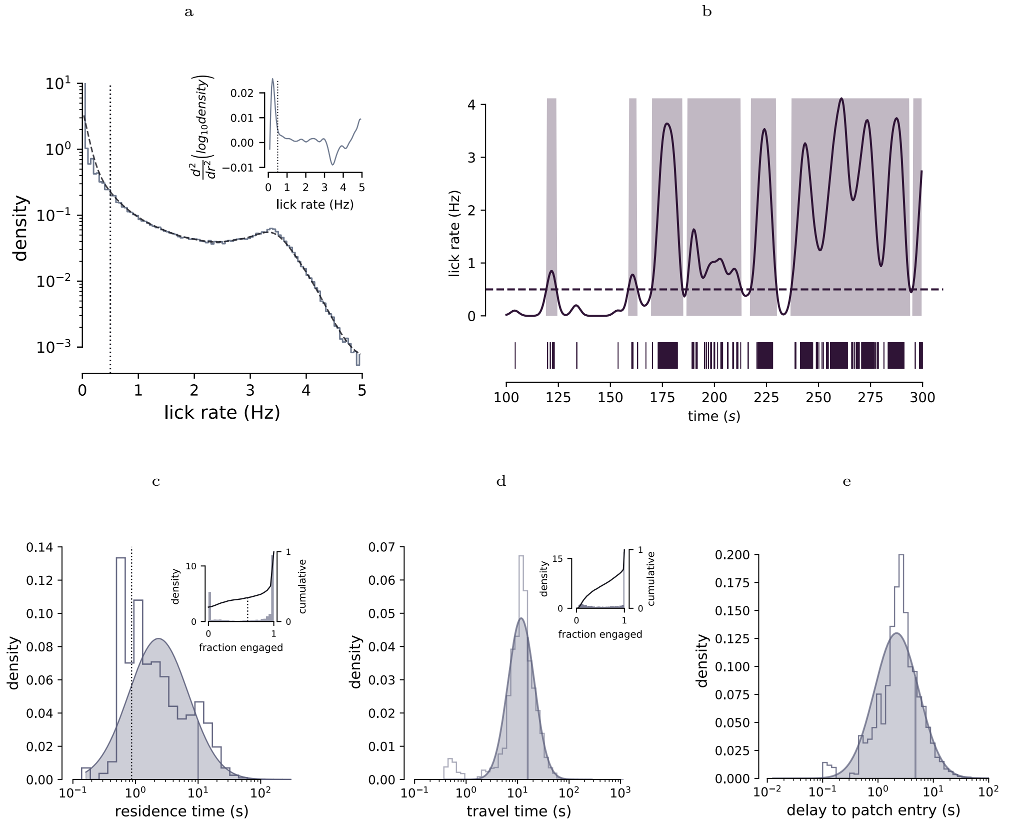


Figure S4: Inclusion criteria for the head-fixed task. (**A**) The raw (solid line) and smoothed (dotted curve) histogram of the smoothed lick rate (bin size = 0.5 seconds, $\sigma$ = 2 seconds) is shown for data pooled from all animals ($N$ = 3) on the head-fixed task. The rate threshold for active engagement (vertical dotted line) was chosen to represent the "elbow" of the second derivative of the smoothed histogram of lick rates (*inset*). (**B**) The animal licks (raster at bottom) and smoothed lick rate (solid purple curve) are shown for a 200-second window of an example session. The residence time (shaded areas) was estimated as the time during which the smoothed lick rate exceeded the rate threshold (horizontal dotted line). (**C-E**) A histogram (step-wise solid line) and fitted log-normal distribution (shaded area) with its associated median (vertical solid line) are shown for the estimated residence times (**C**; calculated per **B**), travel times (**D**; calculated as time between patches during which velocity exceeded 0.5 cm/s), and delay from traveling the required distance to stopping within the next patch (**E**). (*insets*) Histograms (purple bins, left axis) and cumulative summations (solid line, right axis) of the fraction of raw residence (**C**) and travel (**D**) times during which the animal met the respective engagement criteria. Residence times additionally required at least 60% engagement (vertical dotted line in **C**) for the patches to be included in the analysis.

bioRxiv preprint doi: https://doi.org/10.1101/2024.03.30.587253; this version posted March 31, 2024. The copyright holder for this preprint (which was not certified by peer review) is the author/funder, who has granted bioRxiv a license to display the preprint in perpetuity. It is made available under aCC-BY 4.0 International license.

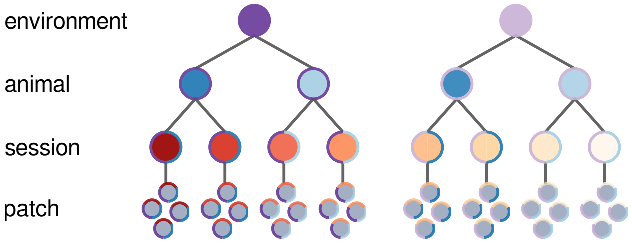


Figure S5: Overview of the cluster bootstrap approach. Residence times, which are the data points of analysis, exist within a hierarchical structure of contextual characteristics that influence outcomes, including the environment (such as travel distance or reward decay rate), animal, or session in which the patch occurred. When sampling via the bootstrap method, these characteristics (colored outlines of circles) must be appropriately randomized at each level to respect their individual contributions to the overall outcomes.

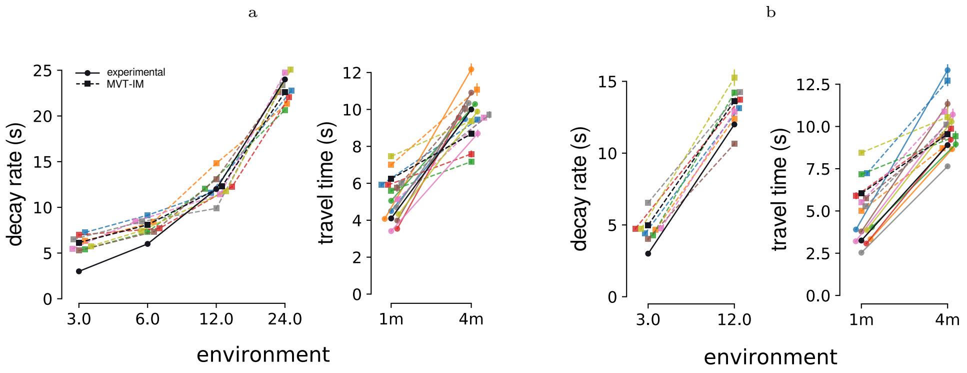


Figure S6: Internal models of environmental parameters. For environments with low- (**A**; $RSI = 0.1$) and moderate-to-high (**B**; $RSI \in [0.5, 1.0]$) stochasticity, the estimates of the reward decay rate (left) and travel time (right) were calculated per the MVT-IM model, which constrained each animal to have a one-to-one mapping between parameter estimates (vertical axis) and unique environments (horizontal axis). Internal estimates are shown for individual animals (colored squares and dotted lines), which were used in the analysis, and for pooled data (black squares and dotted lines), which are shown for visualization purposes. The experimental values are also shown for individual animals (colored circles and solid lines) and pooled data (black circles and solid lines); note that individual experimental decay rates were equivalent (i.e. independent of animal behavior) and thus are not shown.

bioRxiv preprint doi: https://doi.org/10.1101/2024.03.30.587253; this version posted March 31, 2024. The copyright holder for this preprint (which was not certified by peer review) is the author/funder, who has granted bioRxiv a license to display the preprint in perpetuity. It is made available under aCC-BY 4.0 International license.

| RSI | parameter | $\beta$ | $\chi^2$ | $p$ |
|---|---|---|---|---|
| 0.05 | $\tau$ | 7.14 [ 6.88 , 7.39 ] | 2649.38 | <0.001 |
| | $\hat{t}^{(t)}$ | 1.10 [ 0.84 , 1.36 ] | 67.13 | <0.001 |
| | $n_p$ | −4.71 [−5.18 , −4.23 ] | 366.52 | <0.001 |
| 1.0, 2.0 | $\tau$ | 5.35 [ 5.08 , 5.61 ] | 1354.05 | <0.001 |
| | $\hat{t}^{(t)}$ | 0.84 [ 0.36 , 1.31 ] | 12.07 | <0.001 |
| | $n_p$ | −5.65 [−6.35 , −4.94 ] | 238.01 | <0.001 |

Table S1: Linear mixed-effects models of the freely moving task. Parameters were fit as predictors of residence time for patches in low-stochasticity ($RSI = 0.05$, $m = 9547$ patches) or high-stochasticity ($RSI \in [1.0, 2.0]$, $m = 4513$ patches) environments. All fixed effects were normalized to the range [0, 1]. Coefficient values are provided as mean [95% CI]. $\chi^2$ and $p$ values were generated from likelihood ratio tests between the full model and reduced model with the respective parameter removed. Key: $\tau$ = decay rate, $\hat{t}^{(t)}$ = task-relevant travel time, $n_p$ = patch number.

| RSI | parameter | $\beta$ | $\chi^2$ | $p$ |
|---|---|---|---|---|
| 0.05 | $\tau$ | 8.49 [ 7.32 , 9.65 ] | 188.67 | <0.001 |
| | $\hat{t}^{(t)}$ | −1.30 [−2.71 , 0.11 ] | 3.24 | 0.072 |
| | $n_p$ | −3.03 [−5.06 , −1.00 ] | 7.50 | 0.006 |
| 1.0, 2.0 | $\tau$ | 1.53 [ 0.47 , 2.58 ] | 7.95 | 0.005 |
| | $\hat{t}^{(t)}$ | 6.30 [ 4.73 , 7.88 ] | 59.11 | <0.001 |
| | $n_p$ | −2.90 [−5.23 , −0.56 ] | 5.89 | 0.015 |

Table S2: Linear mixed-effects models of the head-fixed task. Notation and analysis follows Table S1. Low-stochasticity ($RSI = 0.05$, $m = 1299$ patches) and high-stochasticity ($RSI \in [1.0, 2.0]$, $m = 787$ patches) environments were analyzed separately.

| task | RSI | decay rate ($\tau$) | | | | track length | | |
|---|---|---|---|---|---|---|---|---|
| freely-moving | 0.05 | 3 | 6 | 12 | 24 | | 1.0 | 4.0 |
| | 0.50 | 3 | | 12 | | | 1.0 | 4.0 |
| | 1.00 | 3 | | 12 | | | 1.0 | 4.0 |
| head-fixed | 0.05 | 3 | 6 | 12 | | 0.6 | 1.0 | 2.0 |
| | 0.50 | 3 | | 12 | | | 1.0 | 2.0 |
| | 1.00 | 3 | | 12 | | | 1.0 | 2.0 |

Table S3: List of Environmental Parameters. Reward stochasticity index (RSI) and decay rate are defined elsewhere. Decay rate is given in seconds and track length in meters.
